# Harmonizing definitions for hematopoietic recovery, graft rejection, graft failure, poor graft function, and donor chimerism in allogeneic hematopoietic cell transplantation: a report on behalf of the EBMT, ASTCT, CIBMTR, and APBMT

**DOI:** 10.1038/s41409-024-02251-0

**Published:** 2024-03-05

**Authors:** Anna Sureda, Paul A. Carpenter, Andrea Bacigalupo, Vijaya Raj Bhatt, Josu de la Fuente, Aloysius Ho, Leslie Kean, Jong Wook Lee, Isabel Sánchez-Ortega, Bipin N. Savani, Johannes Schetelig, Edward A. Stadtmauer, Yoshiyuki Takahashi, Yoshiko Atsuta, John Koreth, Nicolaus Kröger, Per Ljungman, Shinichiro Okamoto, Uday Popat, Robert Soiffer, Heather E. Stefanski, Mohamed A. Kharfan-Dabaja

**Affiliations:** 1 Clinical Hematology Department, Institut Català d’Oncologia-L’Hospitalet, IDIBELL, Universitat de Barcelona, Barcelona, Spain; 2 Clinical Research Division, Fred Hutchinson Cancer Center, Seattle, WA USA; 3 Fondazione Policlinico Universitario A. Gemelli IRCCS, Roma, Italy; 4 Fred & Pamela Buffett Cancer Center, University of Nebraska Medical Center, Omaha, NE USA; 5 Department of Paediatrics, Imperial College Healthcare NHS Trust, St Mary’s Hospital, London, UK; 6 Department of Immunology & Inflammation, Imperial College London, London, UK; 7 Department of Haematology, Singapore General Hospital and National Cancer Centre Singapore, Singapore, Singapore; 8 Stem Cell Transplantation Program. Division of Hematology/Oncology, Boston Children’s Hospital, Boston, MA USA; 9 Department of Pediatric Oncology, Dana-Farber Cancer Institute, Boston, MA USA; 10 Division of Hematology, Seoul St. Mary’s Hospital, The Catholic University of Korea, Seoul, Republic of Korea; 11 EBMT Medical officer, EBMT executive office, Barcelona, Spain; 12 Division of Hematology/ Oncology, Department of Medicine, Vanderbilt University Medical Center, Nashville, TN USA; 13 Medical Department I, University Hospital Carl Gustav Carus. TU Dresden & DKMS Group, Clinical Trials Unit, Dresden, Germany; 14 Perelman School of Medicine, University of Pennsylvania, Philadelphia, PA USA; 15 Department of Pediatrics, Nagoya University Graduate School of Medicine, Aichi, Japan; 16 Japanese Data Center for Hematopoietic Cell Transplantation, Nagakute, Japan; 17 Department of Registry Science for Transplant and Cellular Therapy, Aichi Medical University School of Medicine, Nagakute, Japan; 18 Department of Medical Oncology, Division of Hematologic Malignancies, Dana-Farber Cancer Institute, Boston, MA USA; 19 Department for Stem Cell Transplantation, University Medical Center Hamburg, Hamburg, Germany; 20 Department. of Cellular Therapy and Allogeneic Stem Cell Transplantation. Karolinska Comprehensive Cancer Center, Karolinska University Hospital Huddinge, Division of Hematology, Department of Medicine Huddinge, Karolinska Institutet, Stockholm, Sweden; 21 Division of Hematology, Department of Medicine, Keio University School of Medicine, Tokyo, Japan; 22 Department of Stem Cell Transplantation & Cellular Therapy, The University of Texas MD Anderson Cancer Center, Houston, TX USA; 23 Center for International Blood and Marrow Transplant Research, National Marrow Donor Program/Be The Match, Minneapolis, MN USA; 24 Division of Hematology-Oncology and Blood and Marrow Transplantation and Cellular Therapy Program, Mayo Clinic, Jacksonville, FL USA

**Keywords:** Health care, Diseases

## Abstract

Despite emergence of novel therapies to treat hematologic malignancies, allogeneic hematopoietic cell transplantation (allo-HCT) remains an essential treatment modality capable of curing these diseases. Allo-HCT has been also shown to be curative in benign hematologic disorders such as aplastic anemia, sickle cell disease, and thalassemia, among others. Recently, the American Society for Transplantation and Cellular Therapy (ASTCT) published standardized definitions for hematopoietic recovery, graft rejection, graft failure, poor graft function, and donor chimerism. To attempt broader international consensus, a panel of adult and pediatric physician transplant experts was assembled from European Society for Blood and Marrow Transplantation (EBMT), ASTCT, the Center for International Blood and Marrow Transplant Research (CIBMTR), and Asia-Pacific Blood and Marrow Transplantation (APBMT). Consensus was defined as ≥70% of voting members strongly agreeing or somewhat agreeing with a definition. With few exceptions, there was a consensus to endorse the prior ASTCT definitions. Importantly, we revised existing EBMT and CIBMTR data collection forms to align with these harmonized definitions that will facilitate research and international collaboration among transplant researchers and across transplant registries.

## Introduction

Allogeneic hematopoietic cell transplantation (allo-HCT) remains an essential treatment for malignant and non-malignant hematologic diseases [1–10] with increased utilization being made possible by HCT practice advancements. Examples include increasing success with haploidentical HCT, effectively making a suitable donor now available for over 90% of patients. Publicly banked umbilical cord blood units provide another stem cell source, although hematologic recovery is slower [11–13]. Reduced intensity conditioning (RIC) further broadens allo-HCT feasibility for patients with more advanced age and/or comorbidities that preclude them from receiving more intense conditioning [14–16]. Practice preferences in adults generally favor filgrastim-mobilized peripheral blood stem cells (PBSC) over unstimulated bone marrow cells (BM) due to more convenient procurement and flexibility associated with easier cryopreservation. Each of these advancements potentially can affect engraftment kinetics.

Two crucial events are expected to occur after allo-HCT: engraftment of donor stem cells and appropriate graft function. The first is defined by the proportion of donor cells on chimerism assessment of bone marrow and/or peripheral blood. The second event is hematologic recovery or graft function. Ideally, a patient will achieve full donor chimerism and rapid /complete hematologic recovery, including neutrophils, platelets, and hemoglobin. However, some patients may achieve full donor chimerism with incomplete hematologic recovery and vice versa. These events may occur early, a few weeks posttransplant, or later during long-term follow-up.

Recognizing this complexity, the American Society for Transplantation and Cellular Therapy (ASTCT) recently published standardized definitions of hematopoietic (neutrophil and platelet) recovery, graft rejection, primary and secondary graft failure, poor graft function, and donor chimerism for both pediatric and adult allo-HCT [17]. To attempt broader international consensus, a panel of adult and pediatric physician transplant experts was assembled from European Society for Blood and Marrow Transplantation (EBMT), ASTCT, the Center for International Blood and Marrow Transplant Research (CIBMTR), and Asia-Pacific Blood and Marrow Transplantation Group (APBMT). This project included a second harmonization phase of EBMT and CIBMTR data collection forms to homogeneously capture necessary information for the previously endorsed terms.

## Methods

The EBMT, ASTCT, CIBMTR, and APBMT convened a Steering Committee to address challenging issues in allo-HCT for which definitions vary or are unclear. Each society nominated 3 experts (2 adult physicians and 1 pediatrician) and created a graft failure harmonization of definitions panel.

Based on the definitions of the original publication by Kharfan-Dabaja et al. on behalf of ASTCT [17], expert panel members used a modified Delphi method to facilitate the achievement of a consensus. Panel members voted on all consensus definitions in the ASTCT paper [17] except on definitions for delayed engraftment and primary graft failure after G-CSF stimulated bone marrow, as these statements did not achieve consensus in the original publication (Table 1). Anonymous rating of the statements took place online via SurveyMonkey (by Momentive, San Mateo, CA, USA) according to the five-point Likert scale (strongly agree; somewhat agree; neutral; somewhat disagree; strongly disagree). Consensus was defined as ≥70% of voting members strongly agreeing or somewhat agreeing with a definition. The project manager presented the results of the first rating round during a virtual meeting. Panel members discussed the anonymous ranking of statements and the comments included in the survey. There was also a possibility to re-formulate or to add new statements whenever deemed necessary. Modified statements underwent a second round of voting. Moreover, to ensure that EBMT and CIBMTR data collection forms would be able to capture the required information to define the consensus statements and to harmonize data collection in both registries, a thorough review and side-by-side comparison of EBMT and CIBMTR data collection forms was also performed. Expert panel members discussed the comparison data file and proposed changes for those definitions for which information was not homogeneously or completely captured in the registries.

**Table 1:** Consensus definitions from ASTCT by Kharfan-Dabaja et al. [17].

Term	Definition
Neutrophil recovery	Both panels endorsed the existing definition of neutrophil recovery as the first of 3 successive days with an absolute neutrophil count of ≥500/μL after post-transplantation nadir.
Platelet recovery	Both panels endorsed the definition of platelet recovery as the first of 3 consecutive days with a platelet count of 20,000/μL or higher in the absence of platelet transfusion for 7 consecutive days.
Graft rejection versus graft failure	Both panels defined graft rejection as an immune-mediated process, whereas graft failure represents a wider array of possibilities, including cell dosing, disease, infection, drugs, and an immune-mediated event.
Graft failure (primary)* (according to cell Source)	PBSCs: Both panels defined graft failure as lack of achievement of an ANC ≥500/μL by day +30 with associated pancytopenia.
	Unstimulated BM: Both panels defined graft failure as lack of achievement of an ANC ≥500/μL by day +30 with associated pancytopenia.
	UCB: Both panels defined graft failure as lack of achievement of an ANC ≥500/μL by day +42 with associated pancytopenia.
Poor graft function**	Both panels defined poor graft function as frequent dependence on blood and/or platelet transfusions and/or growth factor support in the absence of other explanations, such as disease relapse, drugs, or infections
Secondary graft failure*	Both panels defined secondary graft failure as a decline in hematopoietic function (may involve hemoglobin and/or platelets and/or neutrophils) necessitating blood products or growth factor support, after having met the standard definition of hematopoietic (neutrophils and platelets) recovery
Donor chimerism	Full: Both panels endorsed the existing definition of full donor chimerism as >95% for both myeloid and lymphoid lineages.
	Mixed or partial: Both panels endorsed the existing definition of mixed donor chimerism as 5%–95% for both myeloid and lymphoid lineages.
	Absent: Both panels endorsed the existing definition of absent donor chimerism as <5% for both myeloid and lymphoid lineages.

Note that delayed engraftment and primary graft failure after G-CSF-stimulated BM were not reviewed nor discussed as no previous consensus was reached in the referenced publication by Kharfan-Dabaja, ASTCT 2021 [17].

*PBSC* peripheral blood stem cells, *ANC* absolute neutrophil count, *BM* bone marrow cells, *G-CSF* granulocyte colony-stimulating factor.

*Donor chimerism testing is also done to confirm the suspicion of graft failure.

**Assumes that donor myeloid and lymphoid chimerism are within a desirable target level.

## Results

### Neutrophil and platelet recovery

As summarized in Table 1, the expert panel members endorsed the existing working definitions of neutrophil and platelet recovery of the previous ASTCT publication [17]. No modifications on the EBMT and CIBMTR data collection forms were required to capture the statements’ information (Supplementary information).

However, for all applicable statements, the expert panel members agreed to use the international system terminology (i.e., 0.5 × 10^9/L for neutrophils and 20 ×1 0^9/L for platelets instead of ≥500/µL and 20,000/µL, respectively).

### Primary graft failure

Expert panel members endorsed the definition of primary graft failure based on the different cell sources (Table 1) [17]. Graft failure statements are agnostic of relapse and assume that donor chimerism testing is also performed to confirm loss of donor chimerism that supports a graft failure diagnosis. No modifications to the EBMT and CIBMTR data collection forms are required to capture the statements’ information (Supplementary information).

### Secondary graft failure

The expert panel members endorsed the definition of secondary graft failure (again agnostic of relapse and assuming that donor chimerism testing is done to confirm the suspicion) (Table 1) [17]. However, after reviewing the pertinent EBMT and CIBMTR data collection forms, the expert panel members recommended question modifications to facilitate capturing the relevant information. Proposals were to remove time constraints by changing early and late graft loss/failure terms to the broader term of secondary graft failure and to include the date of graft failure diagnosis (Supplementary information).

### Donor chimerism

The expert panel members endorsed the current definitions of full donor chimerism as >95% and absent donor chimerism as <5%, for both myeloid and lymphoid lineages (Table 1) [17]. The mixed or partial donor chimerism definition was endorsed in the first round (Table 1) [17]. However, after discussing the diagnostic and therapeutic impact of mixed chimerism that might present in only one of the myeloid or lymphoid subpopulations, the panel agreed to revise the statement to also include those cases where the mixed chimerism (5%–95%) occurs only in one lineage. Thus, a second voting round for the modified statement was performed and it was then unanimously endorsed (Table 2).

**Table 2:** Mixed or partial donor chimerism definition and proposed modification.

Original statement [Kharfan-Dabaja MA, et al. ASTCT 2021 [17]]	New endorsed statement
Mixed donor chimerism as 5%–95% for both myeloid and lymphoid lineages.	Mixed donor chimerism as 5%–95% for either one or both myeloid and lymphoid lineages

No modifications on the EBMT and CIBMTR data collection forms were required to capture the donor chimerism statements’ information (Supplementary information).

### Graft rejection versus graft failure and poor graft function

The expert panel members endorsed the current definitions set forth in the ASTCT publication (Table 1) [17].

After reviewing the EBMT and CIBMTR data collection forms questions, the expert panel members recommended to modify some questions to be able to correctly capture the information. Proposals were to change the “rejection/poor graft function or failure” terms as cause of death and indication for subsequent allo-HCT to “graft failure or poor graft function” (Supplementary information). Moreover, to be able to capture information on the immune-mediated process, the proposal was to include in both registries’ data collection forms the question “were donor-specific antibodies identified?” with “yes” (and date of first detection), “no” and “not done” answers (Supplementary information).

## Case reports

Three cases are provided to illustrate the real-world applicability of the more complex consensus definitions, specifically “poor graft function “primary *versus* secondary graft failure” and “mixed chimerism” after HCT for hemoglobinopathy.

### Case #1. Poor graft function

A 47-year-old man received an HLA-matched allogeneic HCT from a 43-year-old unrelated male donor for favorable risk AML [t(8;21)(q22;q22.1), RUNX1-RUNX1T1] with molecular persistence after multiple chemotherapy treatment lines. Myeloablative conditioning comprised intravenous busulfan (3.2 mg/kg on each of days −7 to −4) and intravenous cyclophosphamide (60 mg/kg on each of days −3 to −2). Cell source was PBSC (3.7 × 10^6^ CD34^+^/kg). Graft-vs.-host-disease (GVHD) prophylaxis was cyclosporine A, short course methotrexate, and mycophenolate mofetil (MMF). Neutrophils and platelets recovered at day +25 and +28, respectively. Two months after transplant the WBC was ~2.0 × 10^9^/L with ANC ~ 1.1 × 10^9^/L but he still required weekly packed red blood cell (PRBC) transfusions and twice weekly platelet transfusions to maintain hemoglobin >8.0 g/dL and platelets >20 × 10^9^/L. Serum ferritin was already 7341 ng/mL early after transplant secondary to frequent red cell transfusion support during pre-transplant chemotherapy courses. He was on 500 mg/12 h MMF as immunosuppressive treatment.

Bone marrow re-staging at days +30 and +60 showed patchy hematopoiesis with overall cellularity of 5% but complete remission of AML was confirmed by flow cytometry and molecular testing together with 100% donor chimerism in unsorted marrow, 99% CD33, and 95% CD3 in sorted peripheral blood. He had no overt clinical signs of infection and serial viral PCR tests in peripheral blood and marrow were also negative; no signs or symptoms of GVHD, so immunosuppressive treatment was withdrawn as initially planned.

*This case of poor hematologic recovery 60 days after transplantation, in complete remission, full donor chimerism, and in the absence of other explanations (disease relapse, drug toxicity, infections, GVHD…) represents a case of poor graft function. Potential contributing causes include the use of a non-sibling donor, low CD34*^*+*^ *cell dose and iron overload*.

### Case #2. Primary graft failure versus secondary graft failure

A 45-year-old man received a 9/10 HLA-matched unrelated donor allogeneic HCT for high-risk AML with 5% blasts at time of HCT. He was conditioned with high-dose melphalan, fludarabine, and 8-Gy total body irradiation. Cell source was PBSC (6.8 × 10^6^ CD34 + /kg). GVHD prophylaxis was posttransplant cyclophosphamide and tacrolimus. Neutrophil and platelet recovery occurred on days +24 and +32, respectively. At day +30 the marrow showed persistent disease with 3% blasts. Donor chimerism was 95% for both T cell and myeloid sorted peripheral blood leukocyte fractions as well as for unsorted bone marrow. By day +35, he developed refractory CMV reactivation on high-dose ganciclovir which preceded the decrease in neutrophil counts and hemoglobin levels. He received filgrastim and PRBC transfusions every 5 days to maintain a hemoglobin >8.0 g/dL. Bone marrow re-staging on day +60 showed persistent leukemia with 4% blasts at which time peripheral blood chimerism analysis showed donor T cells down to 25%, donor granulocytes down to 75%, while unfractionated marrow showed 85% return of host hematopoiesis (only 15% donor).

*The evidence of neutrophil and platelet recovery and full donor chimerism on day* + *35 excludes primary graft failure. However, the significant decrease in hematopoietic function plus loss of full donor chimerism at day* + *60 is most consistent with the secondary graft failure diagnosis, possibly precipitated by CMV reactivation, treatment with ganciclovir, and leukemic persistence*.

### Case #3. Figure 1 – Mixed chimerism in a patient with sickle cell disease

**Fig. 1:**
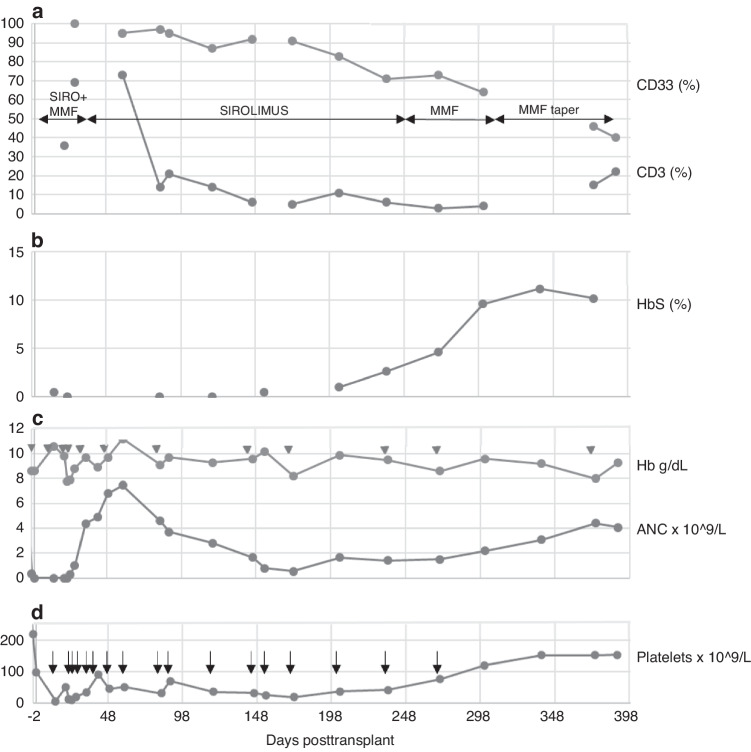
Mixed chimerism in a patient with sickle cell disease. **A** CD33 and CD3 percentages during the post-transplant period. **B** HbS percentage during the post-transplant period. **C** Hemoglobin (Hb) and absolute neutrophil counts (ANC) during the post-transplant period. Triangles show red cell transfusions. **D** Platelets counts during the post-transplant period. Arrows show platelets transfusions.

A 39-year-old female was conditioned with Astatine^211^-anti-CD45 monoclonal antibody, fludarabine (150 mg/m^2^), cyclophosphamide (29 mg/kg), thymoglobulin (4.5 mg/kg), 3 Gy total body irradiation and then received an HLA-matched PBSC transplant from an unrelated donor. Posttransplant cyclophosphamide (50 mg/kg) was given on D + 3 and D + 4; MMF and sirolimus began on D + 5. Recovery of neutrophils, platelets, and 100% donor hemoglobin (absence of HbS) occurred by D + 30 but platelet transfusions (*arrows in* Fig. 1) were needed until 8 months post-HCT (*red cell transfusions shown by triangles in* Fig. 1). However, from D + 84, peripheral blood chimerism analysis revealed a precipitous decline in donor T cells (CD3) and a slower decline in donor granulocytes (CD33), with reemergence of host HbS beginning from D + 154. Augmentation of systemic immunosuppression did not reverse falling donor chimerism. After D + 301, the alternative approach of immunosuppression withdrawal was attempted. From D + 375, although donor T cells appear to be rising, donor granulocytes continue to decline, consistent with impending secondary graft failure.

*This case underscores the relevance of trending sorted peripheral blood leukocyte donor chimerism and HbS in a case of sickle cell disease where CBC monitoring alone is insufficient for understanding of graft durability and graft function*.

## Discussion

This project represented an effort by three major HCT societies, EBMT, ASTCT, and APBMT, together with the CIBMTR registry, to harmonize recently established definitions of hematopoietic recovery, graft rejection, graft failure, poor graft function, and donor chimerism in allo-HCT for broader applicability [17]. One aspect unique to this effort is that we also revised existing data collection forms of the two major HCT registries, namely EBMT and CIBMTR, to align with these definitions.

With few minor exceptions, there was consensus to endorse ASTCT definitions [17]. These exceptions included using the International System terminology; for instance, 0.5 × 10^9^/L for neutrophils and 20 × 10^9^/L for platelets, instead of ≥500/µL and 20,000/µL, respectively as per ASTCT publication [17]. The other exception was pertaining to definition of partial (or mixed) donor chimerism as 5%–95% for either “one or both” myeloid and lymphoid lineages rather than both lineages as originally described [17].

The harmonization committee also recognized that although consensus was reached for the graft failure definition, operationally this definition (which is agnostic of relapse) could feel limiting when relapse was judged clinically to be solely responsible for the graft failure. Committee members generally deemed this scenario as “relapse”, implicitly stating that “relapse” is mutually exclusive of “graft failure”. However, in practice, the potential contributors to graft failure likely interact on a continuum. For example, most clinicians would classify pancytopenia, falling donor chimerism, with 90% leukemia blasts, as “relapse” and not “graft failure”. However, cytopenias, falling donor chimerism with 2% leukemia blasts in the context of concomitant marrow suppressive infections, medications, or other processes (e.g., thrombotic microangiopathy) is less straightforward. Operationally, our harmonized definition allows for real-world nuance by recognizing that relapse may not always be the predominant cause of graft failure. Since registries collect discrete relapse data in parallel, a study of posttransplant outcomes that uses the harmonized graft failure definition should be able to report not only complete relapse rates, but also primary and secondary graft failure rates, as well as whether relapse was or was not a contributor to graft failure.

We believe that this work will help disseminate this information to a larger number of transplant physicians in Europe, Asia, and Australia and facilitate further collaborations among major transplant registries worldwide.

This project did not intend to prescribe recommendations for managing disease relapse and/or partial (or mixed) or absent donor chimerism. Also, we direct the readers to the ASTCT publication [17] regarding the optimal time point(s) to measure donor chimerism as this clinical question was not addressed in the current effort.

### Supplementary information

EBMT-CIBMTR statements data collection comparison.

## Data Availability

Data sharing not applicable to this article as no datasets were generated or analyzed during the current study.
