# Correction: Harmonizing definitions for hematopoietic recovery, graft rejection, graft failure, poor graft function, and donor chimerism in allogeneic hematopoietic cell transplantation: a report on behalf of the EBMT, ASTCT, CIBMTR, and APBMT

**DOI:** 10.1038/s41409-024-02336-w

**Published:** 2024-06-21

**Authors:** Anna Sureda, Paul A. Carpenter, Andrea Bacigalupo, Vijaya Raj Bhatt, Josu de la Fuente, Aloysius Ho, Leslie Kean, Jong Wook Lee, Isabel Sánchez-Ortega, Bipin N. Savani, Johannes Schetelig, Edward A. Stadtmauer, Yoshiyuki Takahashi, Yoshiko Atsuta, John Koreth, Nicolaus Kröger, Per Ljungman, Shinichiro Okamoto, Uday Popat, Robert Soiffer, Heather E. Stefanski, Mohamed A. Kharfan-Dabaja

**Affiliations:** 1 Clinical Hematology Department, Institut Català d’Oncologia-L’Hospitalet, IDIBELL, Universitat de Barcelona, Barcelona, Spain; 2 Clinical Research Division, Fred Hutchinson Cancer Center, Seattle, WA USA; 3 Fondazione Policlinico Universitario A. Gemelli IRCCS, Roma, Italy; 4 Fred & Pamela Buffett Cancer Center, University of Nebraska Medical Center, Omaha, NE USA; 5 Department of Paediatrics, Imperial College Healthcare NHS Trust, St Mary’s Hospital, London, UK; 6 Department of Immunology & Inflammation, Imperial College London, London, UK; 7 Department of Haematology, Singapore General Hospital and National Cancer Centre Singapore, Singapore, Singapore; 8 Stem Cell Transplantation Program. Division of Hematology/Oncology, Boston Children’s Hospital, Boston, MA USA; 9 Department of Pediatric Oncology, Dana-Farber Cancer Institute, Boston, MA USA; 10 Division of Hematology, Seoul St. Mary’s Hospital, The Catholic University of Korea, Seoul, Republic of Korea; 11 EBMT Medical officer, EBMT executive office, Barcelona, Spain; 12 Division of Hematology/ Oncology, Department of Medicine, Vanderbilt University Medical Center, Nashville, TN USA; 13 Medical Department I, University Hospital Carl Gustav Carus. TU Dresden & DKMS Group, Clinical Trials Unit, Dresden, Germany; 14 Perelman School of Medicine, University of Pennsylvania, Philadelphia, PA USA; 15 Department of Pediatrics, Nagoya University Graduate School of Medicine, Aichi, Japan; 16 Japanese Data Center for Hematopoietic Cell Transplantation, Nagakute, Japan; 17 Department of Registry Science for Transplant and Cellular Therapy, Aichi Medical University School of Medicine, Nagakute, Japan; 18 Department of Medical Oncology, Division of Hematologic Malignancies, Dana-Farber Cancer Institute, Boston, MA USA; 19 Department for Stem Cell Transplantation, University Medical Center Hamburg, Hamburg, Germany; 20 Department. of Cellular Therapy and Allogeneic Stem Cell Transplantation. Karolinska Comprehensive Cancer Center, Karolinska University Hospital Huddinge, Division of Hematology, Department of Medicine Huddinge, Karolinska Institutet, Stockholm, Sweden; 21 Division of Hematology, Department of Medicine, Keio University School of Medicine, Tokyo, Japan; 22 Department of Stem Cell Transplantation & Cellular Therapy, The University of Texas MD Anderson Cancer Center, Houston, TX USA; 23 Center for International Blood and Marrow Transplant Research, National Marrow Donor Program/Be The Match, Minneapolis, MN USA; 24 Division of Hematology-Oncology and Blood and Marrow Transplantation and Cellular Therapy Program, Mayo Clinic, Jacksonville, FL USA

**Keywords:** Health care, Diseases

Correction to: *Bone Marrow Transplantation* 10.1038/s41409-024-02251-0, published online 5 March 2024

The Funding information section was missing from this article and should have read:

CIBMTR is supported primarily by the Public Health Service U24CA076518 from the National Cancer Institute (NCI), the National Heart, Lung and Blood Institute (NHLBI), and the National Institute of Allergy and Infectious Diseases (NIAID); 75R60222C00011 from the Health Resources and Services Administration (HRSA); and N00014-23-1-2057 and N00014-24-1-2057 from the Office of Naval Research. Support is also provided by the Medical College of Wisconsin, NMDP, Gateway for Cancer Research, Pediatric Transplantation and Cellular Therapy Consortium and from the following commercial entities: AbbVie; Actinium Pharmaceuticals, Inc.; Adaptive Biotechnologies Corporation; ADC Therapeutics; Adienne SA; Alexion; Allogene; AlloVir, Inc.; Amgen, Inc.; Astellas Pharma US; AstraZeneca; Atara Biotherapeutics; BeiGene; BioLineRX; Blue Spark Technologies; bluebird bio, inc.; Blueprint Medicines; Bristol Myers Squibb Co.; CareDx Inc.; CSL Behring; CytoSen Therapeutics, Inc.; DKMS; Eurofins Viracor, DBA Eurofins Transplant Diagnostics; Gamida-Cell, Ltd.; Gift of Life Biologics; Gift of Life Marrow Registry; GlaxoSmithKline; HistoGenetics; Incyte Corporation; Iovance; Janssen Research & Development, LLC; Janssen/Johnson & Johnson; Jasper Therapeutics; Jazz Pharmaceuticals, Inc.; Karius; Kashi Clinical Laboratories; Kiadis Pharma; Kite, a Gilead Company; Kyowa Kirin; Labcorp; Legend Biotech; Mallinckrodt Pharmaceuticals; Med Learning Group; Merck & Co.; Mesoblast; Millennium, the Takeda Oncology Co.; Miller Pharmacal Group, Inc.; Miltenyi Biotec, Inc.; MorphoSys; MSA-EDITLife; Neovii Pharmaceuticals AG; Novartis Pharmaceuticals Corporation; Omeros Corporation; OptumHealth; Orca Biosystems, Inc.; OriGen BioMedical; Ossium Health, Inc.; Pfizer, Inc.; Pharmacyclics, LLC, An AbbVie Company; PPD Development, LP; REGiMMUNE; Registry Partners; Rigel Pharmaceuticals; Sanofi; Sarah Cannon; Seagen Inc.; Sobi, Inc.; Stemcell Technologies; Stemline Technologies; STEMSOFT; Takeda Pharmaceuticals; Talaris Therapeutics; Vertex Pharmaceuticals; Vor Biopharma Inc.; WellSky; Xenikos BV.

The original article has been corrected.

