# Supplementary material for: Harmonizing definitions for hematopoietic recovery, graft rejection, graft failure, poor graft function, and donor chimerism in allogeneic hematopoietic cell transplantation: a report on behalf of the EBMT, ASTCT, CIBMTR, and APBMT
Source: Bone Marrow Transplant. 2024 Mar 5;59(6):832–7. doi: 10.1038/s41409-024-02251-0 (PMC11161398; doi:10.1038/s41409-024-02251-0)
Supplement: Supplementary file 1 — EBMT-CIBMTR statements data collection comparison. [file 41409_2024_2251_MOESM1_ESM.docx]

**SUPPLEMENTARY INFORMATION**

For all applicable statements, the expert panel members agreed to change the international system units (SI) terminology (i.e. 0.5x10^9/L for neutrophils and 20x10^9/L for platelets instead of ≥500/µL and 20,000/µL, respectively).

**Neutrophil recovery**

*Both panels endorsed the existing definition of neutrophil recovery as the first of 3 successive days with an absolute neutrophil count of ≥500/µL after post-transplantation nadir.*

No modifications on the EBMT and CIBMTR data collection forms were required to capture the statements’ information.

**Questions from EBMT to answer the statement**

| Absolute neutrophil count (ANC) recovery (Neutrophils ≥ 0.5x10^9/L; first of 3 consecutive values after 7 days without any transfusion containing neutrophils) |
| --- |
| - - - - - No. Date of last assessment |
| - - - - - Yes. Date of ANC recovery |
| - - - - - Never below |
| - - - - - Unknown |

**Questions from CIBMTR to answer the statement**

| Was there evidence of initial ANC hematopoietic recovery? | | | |
| --- | --- | --- | --- |
|  | - Yes |  | |
|  | - No |  | |
|  | - NA |  | |
|  | - Previously reported | |  |
| Date ANC ≥ 500/mm^3 (first of 3 lab values) | | | |

**Platelet recovery**

*Both panels endorsed the definition of platelet recovery as the first of 3 consecutive days with a platelet count of 20,000/µL or higher in the absence of platelet transfusion for 7 consecutive days*

No modifications on the EBMT and CIBMTR data collection forms were required to capture the statements’ information

**Questions from EBMT to answer the statement**

| Platelet reconstitution (Platelets ≥ 20x10^9/L; first of 3 consecutive values after 7 days without transfusion) | | | |
| --- | --- | --- | --- |
|  | - No: Date of last assessment |  | |
|  | - Yes: Date of platelet reconstitution | |  |
|  | - Date unknown |  | |
|  | - Never below | | |
|  | - Unknown |  | |

**Questions from CIBMTR to answer the statement**

| Was an initial platelet count ≥ 20×10^9^/L achieved? |
| --- |
| - Yes |
| - No |
| - NA |
| - Previously reported |
| Date platelet ≥ 20×10^9^/L |

**PRIMARY GRAFT FAILURE**

**Primary Graft failure PBSC**

*Both panels defined graft failure as lack of achievement of an ANC ≥500/uL by day +30 with associated pancytopenia. (Donor chimerism testing is also done to confirm the suspicion of graft failure).*

**Primary Graft failure Unstimulated BM**

*Both panels defined graft failure as lack of achievement of an ANC ≥500/uL by day +30 with associated pancytopenia. (Donor chimerism testing is also done to confirm the suspicion of graft failure).*

**Primary Graft failure UCB**

*Both panels defined graft failure as lack of achievement of an ANC ≥ 500/uL by day +42 with associated pancytopenia (Donor chimerism testing is also done to confirm the suspicion of graft failure).*

No modifications on the EBMT and CIBMTR data collection forms were required to capture the statements’ information

**Questions from EBMT to answer the statement**

| ANC recovery (Neutrophils ≥ 0.5x10^9/L; first of 3 consecutive values after 7 days without any transfusion containing neutrophils) |
| --- |
| - - - - - No. Date of last assessment |
| - - - - - Yes. Date of ANC recovery |
| - - - - - Never below |
| - - - - - Unknown |

| Platelet reconstitution (Platelets ≥ 20x10^9/L; first of 3 consecutive values after 7 days without transfusion) | | | |  |
| --- | --- | --- | --- | --- |
|  | - No: Date of last assessment |  | | |
|  | - Yes: Date of platelet reconstitution | |  | |
|  | - Date unknown |  | | |
|  | - Never below | | | |
|  | - Unknown |  | | |

**Questions from CIBMTR to answer the statement:**

| Was there evidence of initial ANC hematopoietic recovery? | | | | |
| --- | --- | --- | --- | --- |
|  | - Yes |  | | |
|  | - No |  |  |  |
|  | - NA |  |  |  |
|  | - Previously reported | |  | |
| Date ANC ≥ 500/mm3 (first of 3 lab values) | | | |  |

| Was there evidence of initial hematopoietic recovery?   - Yes - No - NA - Previously Reported | |
| --- | --- |
| Date ANC ≥ 500/mm3 (first of 3 lab values): |  |

| Was an initial platelet count ≥ 20 × 10^9^/L achieved?   - Yes - No - NA - Previously Reported |
| --- |
| Date platelets ≥ 20 × 10^9^/L  **SECONDARY GRAFT FAILURE**  *Both panels defined secondary graft failure as a decline in hematopoietic function (may involve hemoglobin and/or platelets and/or neutrophils) necessitating blood products or growth factor support, after having met the standard definition of hematopoietic (neutrophils and platelets) recovery (Donor chimerism testing is also done to confirm the suspicion of graft failure).*  *DATA COLLECTION FORMS MODIFICATIONS*   - *Remove time constraints 🡪 change Early and Late graft failure/loss to Secondary graft failure/loss.* - *Include the date of secondary graft failure/loss.*   **Modifications for EBMT data collection forms below, in red**   \| Early graft loss/failure (recovery followed by loss of graft within the first 100 days or no recovery at all) 🡪 *modify to: Graft failure and enter date of diagnosis* \| \| \| \| --- \| --- \| --- \| \|  \| - Unknown \|  \| \| - Yes: Type of graft failure - Primary (no recovery at all) - Secondary (after initial recovery) \| \| - Unknown \|  \| Late graft loss 🡪 *modify to: Secondary graft failure and enter date of diagnosis* \| \| \| \| --- \| --- \| --- \| \|  \| - No \|  \| \|  \| - Yes: Date of graft loss \|  \| Graft Loss \| - *modify to: Secondary graft failure and enter date of diagnosis* \| \| \| --- \| --- \| --- \| \|  \| - Yes - No - Not evaluated \|  \|   **Modifications for CIBMTR data collection forms, in red**   \| Did late graft failure occur? --> *modify to: Did secondary graft failure occur? And include date of diagnosis* \| \| --- \| \| - Yes - No \|  \| Following the initial hematopoietic recovery, was there subsequent decline in ANC to < 500/mm^3^ for ≥ 3 days?   - Yes - No   Date of decline in ANC < 500/mm^3^ for ≥ 3 days (first of 3 days that the ANC declined) \| \| --- \| \| Did recipient recover and maintain ANC ≥ 500/mm^3^ following the decline?   - Yes - No   Date of ANC recovery \| |

**DONOR CHIMERISM**

**Full Donor Chimerism**

*Both panels endorsed the existing definition of full donor chimerism as >95% for both myeloid and lymphoid lineages.*

**Absent Donor chimerism**

*Both panels endorsed the existing definition of absent donor chimerism as <5% for both myeloid and lymphoid lineages.*

**Mixed or partial Donor Chimerism** *(New statement endorsed, modification underlined)*

*The subcommittee endorsed the existing definition of mixed donor chimerism as 5% to 95% for either one or both myeloid and lymphoid lineages.*

No modifications on the EBMT and CIBMTR data collection forms were required to capture the statements’ information

**Questions from EBMT to answer the statement**

| Overall chimaerism   - Full donor (>95%) - Patient reconstitution (recipient >95%) - Mixed (partial) - Aplasia - Not informative - Not evaluated |
| --- |
| Indicate the date(s) and results of all tests for all donors  Split the results by donor and by the cell type on which the test was performed if applicable.   - Date of test - ID of donor or CBU given by the centre - Number is the infusion order - cell type on which test was performed and % donor cells - BM - PBMC - T-cells - B-Cells - RBC - Monocytes - PMNs (neutrophils) - Lymphocytes, NOS - Myeloid cells, NOS - Other, specify… - Test used - FISH - Molecular - Cytogenetic - ABO group - Other… - Unknown |

**Questions from CIBMTR to answer the statement:**

| Provide date(s), method(s) and other information for all chimerism studies performed prior to the date of contact  Global Registration Identifier for Donors (GRID)  NMDP cord blood unit ID  Registry donor ID  Non-NMDP cord blood unit ID  Donor date of birth or Donor age  Donor sex   - Male - Female   Date sample collected  Method   - Karyotyping for XX/XY - Fluorescent in situ hybridization (FISH) for XX/XY - Restriction fragment-length polymorphisms (RFLP) - VNTR or STR, micro or mini satellite (also include AFLP) - Other   Specify  Cell source   - Bone marrow - Peripheral blood   Cell type   - Unsorted/whole - Red blood cells - Hematopoietic progenitor cells (CD34+ cells) - Total mononuclear cells (lymphs & monos) - T-cells (include es CD3+, CD4+, and/or CD8+) - B-cells (includes CD19+ or CD20+) - Granulocytes (includes CD33+ myeloid cells) - NK cells (CD56+) - Other   66. Specify  Total cells examined  Number of donor cells  Percent donor cells: % |
| --- |

**Graft rejection versus graft failure**

*Both panels defined graft rejection as an immune-mediated process, whereas graft failure represents a wider array of possibilities, including cell dosing, disease, infection, drugs, and an immune-mediated event.*

*DATA COLLECTION FORMS MODIFICATIONS*

- *Modify rejection/poor graft function or failure as cause of death and indication for subsequent HCT to: graft failure or poor graft function.*
- *To capture information on the immune-mediated process, include a NEW question in both registries: Were Donor-specific antibodies identified? In case of positivity, the first detection date will also be requested.*

**Modifications for EBMT data collection forms below**

*INCLUDE A NEW QUESTION:*

*Were Donor-specific antibodies identified?*

- *Yes (include date of first detection)*
- *No*
- *Not done*

| Contributory cause of death   - Rejection/Poor graft function 🡪 *modify to: Graft failure/Poor graft function* |
| --- |

| Early graft loss (engraftment followed by loss of graft within the first 100 days)  🡪 *modify to: Secondary graft failure and enter date of diagnosis*   - Unknown - No - Yes* | |
| --- | --- |
| Date of graft failure and treatment:   - No - Growth factor - Subsequent transplant - Boost - Other |  |

| Late graft failure 🡪 *modify to: Secondary graft failure and enter date of diagnosis* | | | |
| --- | --- | --- | --- |
|  | - No |  |  |
|  | - Yes: Date of graft failure |  |  |

| Reason for this transplant   - Graft failure after alloHCT |
| --- |

| Graft Loss 🡪 *modify to: Secondary graft failure and enter date of diagnosis*   - No - Yes - Not evaluated |  |
| --- | --- |

**Modifications for CIBMTR data collection forms**

*INCLUDE A NEW QUESTION:*

*Were Donor-specific antibodies identified?*

- *Yes (include date of first detection)*
- *No*
- *Not done*

| Primary and contributing cause of death   - Graft rejection or failure 🡪 *modify to: Graft failure* |  |
| --- | --- |
| What was the indication for subsequent HCT?   - - - Graft failure / insufficient hematopoietic recovery 🡪 *modify to: Graft failure/Poor graft function*     - Persistent primary disease     - Recurrent primary disease     - Planned subsequent HCT, per protocol     - New malignancy (including PTLD and EBV lymphoma)     - Insufficient chimerism     - Other: complete question 10 |  |

| Did late graft failure occur? 🡪 *modify to: Did secondary graft failure occur? And include date of diagnosis* |
| --- |
| - Yes - No |

**Poor graft function**

*Both panels defined poor graft function as frequent dependence on blood and/or platelet transfusions and/or growth factor support in the absence of other explanations, such as disease relapse, drugs, or infections (assumes that donor myeloid and lymphoid chimerism are within a desirable target level).*

*DATA COLLECTION FORMS MODIFICATIONS*

- *As for statement on graft rejection versus graft failure, modify rejection/poor graft function or failure as cause of death and indication for subsequent HCT to: Graft failure or poor graft function.*

**Modifications for EBMT data collection forms**

| Contributory cause of death   - Rejection/Poor graft function 🡪 *modify to: Graft failure/Poor graft function* |  |
| --- | --- |

**Modifications for CIBMTR data collection**

| What was the indication for subsequent HCT?   - - - Graft failure / insufficient hematopoietic recovery 🡪 *modify to: Graft failure/Poor graft function*     - Persistent primary disease     - Recurrent primary disease     - Planned subsequent HCT, per protocol     - New malignancy (including PTLD and EBV lymphoma)     - Insufficient chimerism     - Other |
| --- |
